# Silver-Polystyrene (Ag/PS) Nanocomposites Doped with Polyvinyl Alcohol (PVA)—Fabrication and Bactericidal Activity

**DOI:** 10.3390/nano10112245

**Published:** 2020-11-12

**Authors:** Anna Krzywicka, Elżbieta Megiel

**Affiliations:** Faculty of Chemistry, University of Warsaw, Pasteura 1, 02-093 Warsaw, Poland; krzywaa95@gmail.com

**Keywords:** nanocomposites, silver nanoparticles, polystyrene, nitroxides, antibacterial agents

## Abstract

In the present work, we report the studies on perfectly homogeneous nanocomposites composed of polystyrene-grafted silver nanoparticles (Ag@PS) as a bioactive fulfilment and a mixture of polystyrene (PS) and polyvinyl alcohol (PVA) as a matrix. The procedure developed by our group of the nanocomposites’ preparation consists of three steps: synthesis of narrow-dispersive AgNPs (5.96 ± 1.02 nm); grafting of narrowly dispersed polystyrene onto the surface of AgNPs; thermoforming with a mixture of PS/PVA. Kirby-Bauer (K-B) and Dynamic Shake Flask (DSF) assays revealed high antibacterial activity against a series of Gram(−) and Gram(+) bacteria strains of the fabricated nanocomposites at low silver content (0.5%). We showed that the doping of Ag/PS composites with PVA increases the antibacterial activity of composites. The hydrophilic component in the nanocomposites enables easier water migration inside the polymer matrix, which makes releasing silver nanoparticles and silver ions to the environment facile.

## 1. Introduction

Nanocomposites (NCs) with silver nanoparticles (AgNPs) as fulfilments and polymers as a matrix are intensively studied due to their useful properties resulting from the unique properties of nanosilver and easy processability of polymers. Among a wide range of applications of such materials, the utilization as antibacterial agents is the one most intensively investigated [1]. Although the biocidal activity of silver has been known for centuries, the current rapid development of nanotechnology gives new opportunities in designing biomedical materials containing this element in the form of AgNPs with well-defined composition and structure. Noteworthily, AgNPs exhibit not only a broad antibacterial activity but also antifungal, antiviral, anti-inflammatory, anti-angiogenic, and anti-cancer activities [2]. Whereas, many reports revealed low/relatively low cytotoxicity of AgNPs towards human cells. However, it must be emphasized that the cytotoxicity of nanoparticles depends on many factors. The most important are size, shape, size distribution, surface charge, and kind of the ligands attached on their surface [3,4,5]. Thus, the designing of repeated procedures allowing us to obtain AgNPs with precisely defined morphology and structure is crucial for their biomedical applications. Furthermore, the dispersion of AgNPs in an adequately chosen polymer matrix ensures their long-time stability, enables easy processability of such materials, and allows controllable releasing of the nanoparticles from NCs into the environments [6].

There are three main types of nanoparticle-polymer composites (AgNCs) differing in the structure of the nanoparticles used for their preparation and the manner of their combination with the polymer matrix: (1) AgNPs with non-polymer coating dispersed in the polymer matrix; (2) AgNPs attached to the polymer net, and (3) AgNPs with the polymer-modified surface (core-shell nanostructures) dispersed in the polymer matrix [6,7]. Only in the last case, homogenous composites with a uniform distribution of nanoparticles in a polymer matrix may be achieved [8]. In the current scientific literature, a layer built of polymer chains covalently grafted onto the nanoparticles’ surface is named a polymer brush. Three parameters are crucial for the controlling of AgNPs dispersion in a polymer matrix: (1) grafting density of polymer brush, (2) degree of polymerization of the grafted macromolecules, and (3) degree of polymerization of macromolecules that building the polymer matrix [8]. Additionally, when the structure of matrix polymers and grafted polymers is different, this factor should also be taken into consideration.

In the case of biomedical applications of NCs with AgNPs, the uniform dispersion of fulfilment is crucial because it enables gradual and steady releasing of the antibacterial agent in the form of nanoparticles or silver ions. Noteworthily, the releasing also depends on the polymer matrix affinity to the medium where the NCs are located [9,10,11,12,13].

Great attention is targeted at the AgNCs fabricated from engineering polymers that ensuring their easy processability by typical polymer processing techniques such as injection molding, extrusion, or thermoforming [1].

Polystyrene (PS) is one of the most often commercially used engineering polymers. It is a thermoplastic polymer used in the production of packages, insulation boards, and numerous consumer goods [14,15] as well as medical products such as surgical instruments, dental tools, dressing materials, prosthesis, and many others [16]. For all these applications, uses of PS composites that exhibit biocidal activities, for instance, thanks to the dispersed AgNPs, are highly desirable. However, due to the hydrophobic character of this polymer, the preparation of stable and homogeneous composites requires a proper modification of the nanoparticles’ surface. There are a few reports on the modification of AgNPs with hydrophobic molecules such as oleylamine [17] and polystyrene [18,19] via their physical adsorption onto the nanoparticles’ surface. Although hydrophobic modification of AgNPs’ surface undoubtedly facilitates their dispersion in the hydrophobic matrix, this loosely adsorbed layer cannot efficiently protect the nanoparticles against aggregation during the thermal processing of such materials.

Grafting of polymers onto the surface of nanoparticles means anchoring via covalent bonds (also called polymer brush formation), and it enables the preparation of nanohybrids that are miscible in the polymer matrix and also resistant against aggregation [8].

Recently, our group proposed a novel approach for the preparation of perfectly homogeneous Ag/PS nanocomposites using high-density polystyrene grafted silver nanoparticles. The developed procedure is based on utilizing nitroxide-mediated polymerization (NMRP) and relies on the coupling of growing polystyrene chains with nitroxide-coated silver nanoparticles. These fabricated nanohybrids are readily dispersible in the polystyrene matrix. The prepared composites Ag/PS are thermoforming and exhibit significant antibacterial activity against model Gram(−) (*Pseudomonas aeruginosa*) and model Gram(+) (*Staphylococcus aureus*) pathogenic bacteria. Nevertheless, the antibacterial effect is strong when silver content is relatively high ≥ 2% (*w*/*w*) [20]. Thus, it would be worth to find a means of enabling of decreasing silver content and maintain the same bioactivity. We hypothesized that the introduction into the polystyrene matrix of a hydrophilic polymer admixture would allow for enabling easier silver ion release thanks to more effective water migration inside of the material. Therefore, an excellent antibacterial activity could be achieved for lower silver content. For this purpose, we decided to use poly(vinyl alcohol)—PVA as a hydrophilic admixture for the PS matrix. The PVA is widely used for the fabrication of biomaterials as biocompatible, biodegradable, water-soluble, and at the same time, non-expensive polymers [21,22]. Therefore, the bioactive nanocomposites with PVA as a component can find plenty of interesting applications, among others, in medicine, cosmetics, pharmaceuticals, and the packaging industry.

Very recently, Abdallah and co-workers proved that the nanocomposites with AgNPs as fulfilment and PVA as a matrix exhibit high antibacterial activity against a series of Multi-Drug Resistance bacteria strains [23].

In this work, we present research that reveals the influence of hydrophilic admixture in nanocomposites’ matrix on their antibacterial activity. We developed the procedure allowing us to obtain perfectly homogeneous PS/PVA/Ag nanocomposites containing silver nanoparticles grafted with polystyrene.

Notably, the developed method allows for the preparation of the nanocomposites on a scale of tens of grammes. The developed modification of AgNPs gives them perfect miscibility with the polymer matrix and at the same time high thermal stability. Due to the connection of polystyrene chains via the nitroxide linker with the silver surface, the polymer shell is covalently attached to the nanoparticle. Whereas, the hydrophilic admixture (PVA) allows for gaining efficient antibacterial activity at low silver content.

The prepared nanocomposites with silver content 2%, 1%, and 0.5% were subjected to the comprehensive antibacterial assays towards four strains of Gram(−) type and three strains of Gram(+) using two types of antibacterial assays: the Kirby-Bauer disc susceptibility test and the Dynamic Shake Flask method. The composites fabricated, according to the reported procedure, exhibit high antibacterial activity at silver content as low as 0.5%.

The detailed physicochemical characterizations of the fabricated nanomaterials were carried out using UV-vis spectrophotometry, Size-exclusion chromatography (SEC), Transmission Electron Microscopy (TEM), and Thermogravimetric analysis (TGA).

## 2. Materials and Methods

Styrene (Sigma Aldrich, St. Louis, MO, USA, puriss ≥ 99%) was dried over MgSO_4_ and passed through a basic aluminum oxide column before use (Merck, Kenilworth, NJ, USA, 0.06 mm). Benzoyl peroxide (BPO) was recrystallized twice from the chloroform-methanol mixture and dried in a desiccator; NaBH_4_, AgNO_3_, 4-hydroxy-TEMPO (TEMPOL), PVA (M_w_ = 27 kDa) with a high degree of hydrolysis (98%), and all solvents were purchased from Sigma-Aldrich (St. Louis, MO, USA, puriss ≥ 97%) and used as received.

Bis (*N*-oxy-2,2,6,6-tetramethylpiperidyl)-4,5-dithiooctanoate (DiSS) was synthesized according to the procedure reported in our earlier work [24]. Milli-Q ultrapure water (resistivity 18.2 MΩ cm^−1^, Millipore-Merk, Boston, MA, USA) was used throughout the experiments.

### 2.1. Microbial Strains

Gram(−): *Escherichia coli*—source ATCC 25922, *Salmonella enterica sv Typhimurium*—St11 strain, *Yersinia enterocolitica*—Ye9 strain; *Pseudomonas aeruginosa*—PAO1 strain; *Campylobacter jejuni*—Cj 22 source of the last three strains—Institute of Microbiology collection (University of Warsaw, Faculty of Biology, Warsaw, Poland).

Gram(+): *Staphylococcus aureus*—source ATCC 43300; *Bacillus cereus*—source ATCC 10876; *Listeria monocytogenes*—EGD strain, source—Institute of Microbiology collection (University of Warsaw, Faculty of Biology, Warsaw, Poland).

### 2.2. Techniques

#### 2.2.1. Transmission Electron Microscopy (TEM)

Transmission Electron Microscopy (TEM) analyses were performed using the JEM 1400 JEOL Co. microscope (JEOL, Tokyo, Japan) at 120 kV acceleration voltage.

The solution of the sample (with concentration 1 mg/mL) was cast onto a carbon-coated copper microgrid (200 mesh) and air-dried. For the preparation of the solutions of nanocomposites and AgNPs, THF, and acetone were used, respectively.

#### 2.2.2. UV-vis Spectrophotometry

UV-vis spectra were recorded using a Cary 50 Conc UV/Vis spectrophotometer (Varian, Palo Alto, CA, USA). The spectra were recorded in N, *N*-dimethylformamide (DMF) solutions.

#### 2.2.3. Thermogravimetric Analyses (TGA)

Thermogravimetric (TG) measurements were performed using a TA Instruments DSC Q20 apparatus (TA instruments, New Castle, DE, USA) with thermobalance (precision ± 0.4%; minimal mass 0.02 mg) under an N_2_ atmosphere with scanning rate 10 K min^−1^.

#### 2.2.4. Size-Exclusion Chromatography (SEC)

The Size-Exclusion Chromatography (SEC) analyses were carried out using Waters Alliance 2695 liquid chromatograph equipped with a RID detector (Waters 2414 RI, Milford, MA, USA).

The separations of PSs were performed in tetrahydrofuran (THF) on three columns placed in series: Waters Styragel HR1, HR2, HR4 7.8 × 300 mm (Waters, Milford, MA, USA); The columns were thermostated at 35 °C. HPLC grade THF (Sigma-Aldrich, St. Louis, MO, USA) was used at a flow rate of 0.8 mL min^−1^. The concentration of samples prepared in eluents was 1 mg mL^−1^, 20 µL injections were applied. PS standards were used for calibration (Shodex, Showa Denko K. K., Tokyo, Japan) in the range 1.31 × 10^3^ to 3.64 × 10^6^ Da.

#### 2.2.5. Preparation of Polystyrene Grafted Silver Nanoparticles (Ag@PS)

The first step was the preparation of nitroxide-coated silver nanoparticles (*N*-AgNPs) that can be united with polystyrene macroradicals thanks to the unpaired electrons localized in nitroxide moieties. 

Recently, we developed an efficient protocol for the synthesis of *N*-AgNPs on a scale of tens of micrograms. In this paper, we report a modification of this method, allowing us to fabricate the nanoparticles on a scale of hundreds of milligrams/grams using a lab set composed of the jacketed reactor (with capacity 0.5 L) equipped with a mechanical stirrer connected with the thermostat controlling temperature of the heat/cooling medium in the jacket with an accuracy of ± 0.5 °C in the range −20–130 °C. Figure S1 in the Supplementary Materials shows the photograph of the designed lab set that was used in the preparation of *N*-AgNPS. The developed protocol is as follows: 677.7 mg of DiSS (1.321 mmol) was dissolved in 300 mL of DMF in the reactor, and the mixture was bubbled with argon (for 20 min) and stirred using a mechanical stirrer (1600 rpm); the temperature of the cooling medium was set at −20 °C. When the mixture achieved the set temperature, 1.115 mL of AgNO_3_ solution with concentration 1 M (1.115 mmol) was injected and after 5 min a solution of NaBH_4_ (75.3 mg, 2 mmol) in DMF (120 mL) was added using a peristaltic pump (Thermo Fisher Scientific) with a velocity of pumping 1.5 mL/min. During the addition of the solution of NaBH_4_ stirring of the mixture was accelerated to 3000 rpm. After addition of the reducing agent solution, the mixture became a chocolate-brown color; the stirring was continued with a velocity of 1600 rpm for the next 2 h. Afterwards, the post-reaction mixture was mixed with water (ultra-pure 500 mL), and a pinch of NaCl was added to precipitate the fabricated nanoparticles. The obtained suspension was sonicated (2 min), centrifuged (10,000 rpm, 10 min), the supernatant was discarded, and the precipitate was washed several times profusely with water (ultra-pure). The obtained solid was dried in a vacuum oven at 50 °C for 24 h. Thin-layer chromatography (TLC) confirmed the absence of impurities and not-attached ligands in the solution of the prepared *N*-AgNPs. Finally, the grey, solid product was obtained (160 mg, yield 75%, the yield was recalculated on silver content that was taken from TGA).

The fabricated *N*-AgNPs were used in the preparation of Ag@PSs via coupling with polystyrene macroradicals during nitroxide-mediated polymerization of styrene. The employed procedure was developed very recently by our group [20]. In this paper, we report the succeeded rescaling of this protocol, allowing us to fabricate nanocomposites in gram quantities and their blends with poly(vinyl alcohol) (PVA).

Styrene (45 mL, 392 mmol), TEMPOL (0.409 g, 2.40 mmol) and BPO (0.194 g, 0.80 mmol) were inserted to the Schlenk flask sealed with a silicone rubber septum. The applied molar ratio of reactants St:TEMPOL: BPO was 490:3:1 that allowed for obtaining polystyrene (PS) with a maximum average molecular mass equal to 50.96 kDa (when the monomer conversion reached 100%).

The polymerization mixture was degassed using the four freeze-pump-thaw cycles procedure; next pure and dry argon was introduced to the flask, and the flask was immersed in a thermostatically controlled oil bath, set at 120 °C and placed on a magnetic stirrer. After 24 h from the mixture 5 mL of sample was taken and 20 mL of an acetone solution containing 140 mg *N*-AgNPs was injected into polymerization mixture using a syringe (in the counter flow of argon). Notably, the acetone solution of *N*-AgNPs was degassed for 30 min by dry argon bubbling before the injection.

The polymerization of styrene with *N*-AgNPs was continued for 5 h. After this time, the reaction was stopped by cooling the flask and opening to contact the mixture with air. The post-polymerization mixture was viscous, chocolate-brown, and homogeneous, and any signs of nanoparticles aggregation were not observed.

Silver nanoparticles grafted with polystyrene (Ag@PS) were isolated from the mixture with free polystyrene chains (not attached to the surface) via the precipitation process with n-hexane as the precipitant and THF as a solvent. First, the mixture was dissolved in THF, 5-times excess of n-hexane was added, and the suspension obtained in this way was centrifuged (10,000 rpm, 10 min). The process of purification was repeated until turbidity was not observed after the addition of methanol to the supernatant. The brown-solid product was dried in a vacuum oven for 24 h at 60 °C (a final mass of the product was 520 mg).

#### 2.2.6. Preparation of Nanocomposites PS/PVA/Ag@PS and Their Thermoforming into pastilles

The composites were fabricated using a home-made pellet press die set built of Teflon two disks placed in Teflon cylinder with a proper size channel. Two types of pastilles were prepared: with a diameter of 0.5 cm and mass ca. 30 mg and bigger with diameter 1.5 cm and mass ca. 70 mg. The first of them were used in the Kirby-Bauer disc susceptibility test and the second ones in Dynamic Shake Flask tests.

The polystyrene (PS) used for the NCs preparation was isolated from the sample taken during synthesis Ag@PS before the addition of *N*-AgNPs (see above). SEC analysis performed for this PS sample displayed an average number molecular weight M_n_ = 10.8 kDa, an average molecular weight at the maximum of peak 11.39 kDa, and the polydispersity index equals 1.13.

The appropriate concentration of nanosilver in the pastilles was achieved by mixing Ag@PS with a concentration of Ag 18% (determined from TGA) and proper amounts of PS and PVA to gain, at the same time, 5% of PVA.

The general procedure of the pastilles preparing was the following: a mixture of PS, Ag@PS and PVA in proper proportions and mass 40 mg or 85 mg was ground using a bead mill for 10 min. The prepared powder was placed between two disks inside a channel in the press die set. Afterwards, this mixture was pressed using a laboratory press. The fabricated pastilles were transferred onto a Petri dish, placed on the thermal plate of a magnetic stirrer, and heated at 150 °C. As a result, the pastilles became vitreous, and after cooling them to room temperature, they could be taken out. 

#### 2.2.7. Antibacterial Assays of Ag/PS/PVA

Two types of assay were used to assess the antibacterial activity of the fabricated nanocomposites. Both of them show the ability to release antibacterial agents from solid materials. They differ in their mean of contact of the materials with bacterial strains.

##### Kirby-Bauer Disc Diffusion Susceptibility Test

Kirby-Bauer disc diffusion susceptibility tests were performed according to EUCAST recommendations [25]. The bacterial cultures were diluted to an optical density of 0.5 McFarland standard and swabbed on the surface of Mueller-Hinton agar plates. The pastilles made of composites (Ag/PS/PVA) with the mass 36 ± 5 mg were placed in the agar, and the plates were incubated at 37 °C for 24 h. The zones of inhibition (ZI) were measured as the average from three separate experiments.

##### Dynamic Shake Flask Method (DSFM)

DSF assays were performed in the phosphate buffer in the absence of the nutrient, thus under no bacteria growing conditions.

The flasks containing the phosphate buffer (0.3 M, pH = 7.2) was inoculated with 10^5^ CFU mL^−1^ (colony-forming units per millilitre) bacteria. To each flask, the tablets made of composite (Ag/PS/PVA) with the mass 76 ± 8 mg were placed. The pastilles were not sterilized before contact with the bacteria. The flasks were shaken at 200 rpm for 24 h at 37 °C. Furthermore, analogous cultures were carried out with the tablet made of polystyrene mixed with PVA 5% (*w*/*w*) with the mass 75 ± 5 mg (not sterilized) as a control test. After appropriated periods of contact time, the bacterial concentrations of the microbial suspension were determined by measuring optical density (O.D.) at wavelength 600 nm.

## 3. Results and Discussion

The nanocomposites of polystyrene grafted silver nanoparticles (Ag@PS) with a mixture of polystyrene (PS) and polyvinyl alcohol (PVA) were fabricated. The developed procedure allows for preparing perfectly homogeneous nanomaterials exhibiting antibacterial properties at a very low content of nanosilver (0.5% *w*/*w*).

**Figure 1:**
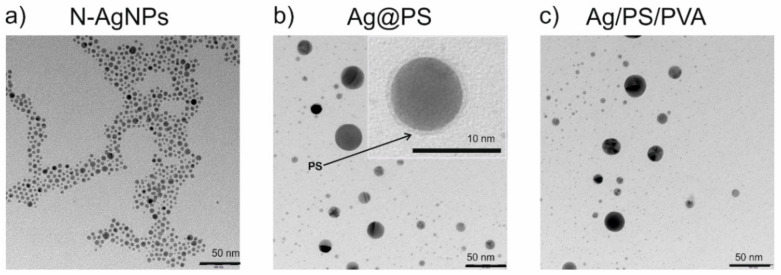
TEM images of the synthesized nanomaterials: nitroxide-coated silver nanoparticles (**a**), polystyrene grafted silver nanoparticles (**b**) and nanocomposites of polystyrene grafted silver nanoparticles with polystyrene doped with polyvinyl alcohol (95:5 *w*/*w* PS:PVA) (**c**).

Figure 1 displays TEM images of nitroxide-coated silver nanoparticles (*N*-AgNPs), the nanohybrids Ag@PS prepared from them, and the fabricated nanocomposites (Ag/PS/PVA).

As can be seen in Figure 1a, the nitroxide-coated silver nanoparticles (*N*-AgNPs) are narrowly dispersed in terms of their size. Further, they have the ability to self-assemble, which is most probably a consequence of interactions between nitroxide radicals and silver. Such interactions cause accumulation of the nanoparticles in partially ordered structures but importantly do not lead to the aggregation. Recently our group reported the existence of the interactions of nitroxide radicals with silver and gold nanoparticles/surfaces [24,26,27,28,29]. 

The presence of nitroxide radicals covalently connected with the silver surface in *N*-AgNPs allows us to connect polystyrene-growing macroradicals during the polymerization and obtain in this way polystyrene grafted silver nanoparticles (Ag@PS). During the polymerization, a fraction of nanoparticles increases in size, as can be seen in Figure 1b. 

**Figure 2:**
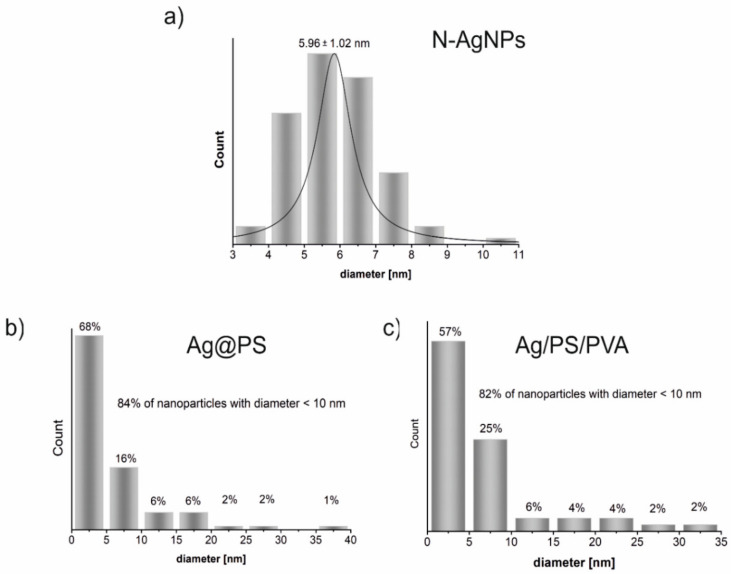
The histograms of the size distribution of silver nanoparticles in the fabricated nanomaterials: nitroxide-coated silver nanoparticles (**a**), polystyrene grafted silver nanoparticles (**b**) and nanocomposites of polystyrene grafted silver nanoparticles with polystyrene doped with polyvinyl alcohol (95:5 *w*/*w* PS:PVA) (**c**). The histograms have been drawn on the base of TEM images shown in Figure 1.

The inset in Figure 1b displays the TEM image of the single silver nanoparticle (magnification with the scale bar 10 nm) where the surrounding polymer shell onto the nanoparticle is visible. Figure 2 displays the histograms drawn on the base of TEM images shown in Figure 1. As can be seen in Figure 2 size distribution of *N*-AgNPs is very narrow, and during the polymerization of styrene with *N*-AgNPs part of nanoparticles increases their size even to above 35 nm, but 84% of the synthesized nanohybrids (Ag@PS) have a diameter below 10 nm. The size distribution is very similar in the nanocomposites fabricated using Ag@PS as fulfilment and the mixture of PS and PVA as a matrix.

TEM studies revealed that the nanocomposites with homogeneously dispersed, non-aggregated spherical silver nanoparticles with the diameter mostly below 10 nm (see Figure 2) were prepared.

Due to the strong localized surface plasmon resonance (LSPR) of silver nanoparticles, the UV-vis spectrophotometry is a very convenient tool to analyze their dispersity in a polymer matrix. The position of LSPR band/bands in the absorption spectrum and its full width at half maximum (FWHM) depend not only on the shape, size, and dispersity of AgNPs, but also on the refractive index of the environment (solvent or solid matrix) [17,30,31].

**Figure 3:**
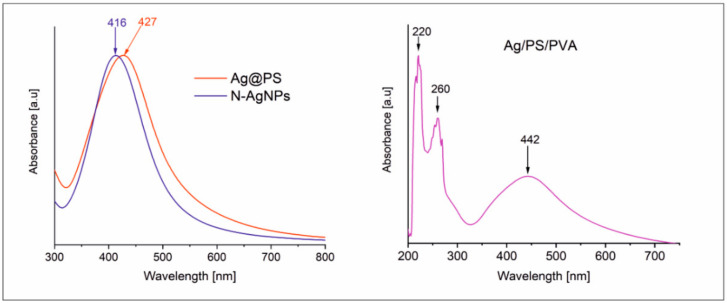
UV-vis absorption spectra recorded for the fabricated nanomaterials (**left**) Ag@PS, N-AgNPs and (**right**) Ag/PS/PVA in DMF solutions at 298 K.

Figure 3 shows the absorption spectra of the prepared silver nanoparticles; polystyrene-grafted nanohybrids and nanocomposites in DMF solutions. The LSPR band in *N*-AgNPs spectrum with a maximum at 416 nm is narrow and symmetric and confirms the narrow dispersity of the nanoparticles. The position of the maximum absorption correlates well with the average size of metal cores ca. 6 nm [28]. For Ag@PS, the LSPR band is red-shifted to 427 nm compared with the band in *N*-AgNPs spectrum. This effect can be explained by changing the refractive index of the protecting layer attached to the surface of the nanoparticles. The maximum of the absorption band for the nanocomposite Ag/PS/PVA is significantly more red-shifted to 442 nm, and also the width of LSPR has increased. It indicates that during their thermal processing, widening of the size dispersity of silver nanoparticles occurs. It is consistent with the results from TEM analyses (see Figure 1 and Figure 2). Notably, the changes in size dispersity are not significant (ca. 80% of nanoparticles retain the same size). In the spectrum of Ag/PS/PVA, two bands characteristic for polystyrene in the UV range at 220 and 260 nm are visible. The absorption bands of PVA in UV-vis spectra are not visible, due to its low concentration in the nanocomposites (5% *w*/*w*). We recorded the spectrum of PVA in the same solvent at the same weight concentration, and any bands were not visible.

The developed procedure for the preparation of Ag@PS is based on the employment of the nitroxide mediated polymerization (NMRP) [32], allowing us to prepare narrowly dispersed polymers with a well-defined molecular weight. Recently, our group developed the method for the grafting of polymer chains on the surface of nanoparticles using NMRP [20,28,29,33]. In this method, nitroxide-coated nanoparticles are injected to the polymerization system controlled by a nitroxide radical. During the polymerization, the growing macroradicals are captured by the radicals attached on the nanoparticles’ surface. In this way, nanohybrid structures with covalently attached polymer chains are obtained (in the case of silver nanoparticles denoted as Ag@PS). Besides the nanohybrids, in the polymerization system, free ones not attached to the nanoparticles polymer chains are prepared with well-defined molecular weights.

Now we use Ag@PS nanohybrids for the fabrication of the Ag/PS/PVA nanocomposites. The polystyrene applied for this purpose (PSF) was separated from the same polymerization system as Ag@PS. The size exclusion chromatography (SEC) was used to determine average molecular weights and dispersity of PSF. Table 1 displays the results of the SEC analysis of PSF. The elugram and molecular weights distribution are presented in the Supplementary Information (Figures S2 and S3, respectively).

Due to the application of the nitroxide radical (TEMPOL) as a mediator, polystyrene formed in the polymerization system (PSF) is narrowly dispersed (polydispersity index PDI = 1.13). In our earlier paper, we proved that the molecular weight of polymers connected with nanoparticles and free in the system is similar. This is so because, during the polymerization, an exchange between attached and free polymer chains is possible [20]. As a consequence, Ag@PS is perfectly miscible in the polymer matrix (PSF) used for the nanocomposites’ (Ag/PS/PVA) preparation. PVA with an averaged molecular weight M_w_ = 27 kDa was used for the nanocomposites’ preparation. Notably, the composites with the concentration of PVA above 5% (*w*/*w*) were becoming non-homogeneous during their thermoforming. Therefore, for all studies, we decided to use only the nanocomposites with 5% PVA.

**Figure 4:**
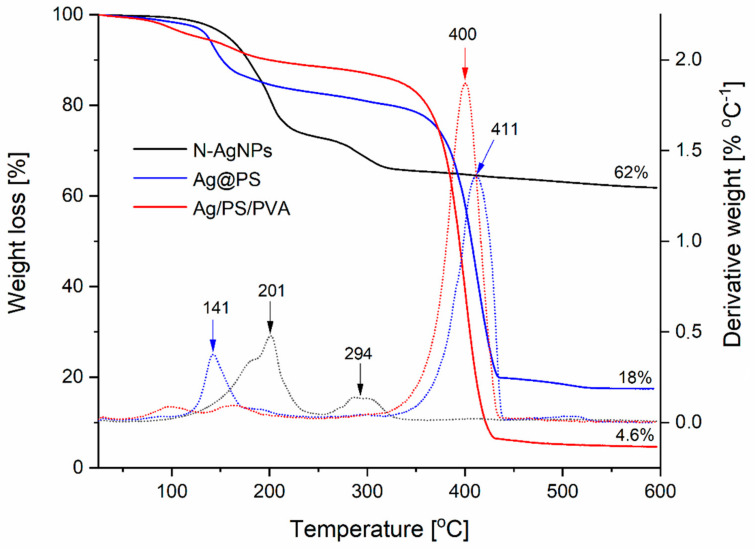
Thermogravimetric analysis (TGA) curves (solid lines) and corresponding first-derivatives of TGA with respect to the temperature (dotted lines with the same colour) of nitroxide-coated silver nanoparticles (*N*-AgNPs), nanohybrids with polystyrene (Ag@PS), their nanocomposites with polystyrene, and polyvinyl alcohol (Ag/PS/PVA). The given above the TGA curves the final percentage of start mass corresponds to silver content in the fabricated materials.

Thermogravimetric analyses (TGA) were conducted to evaluate the stability of the fabricated nanomaterials. Figure 4 shows TGA curves in the range 20–600 °C and the corresponding first derivatives (DTA). 

As can be seen, the decomposition of nitroxide-coated nanoparticles, under the conditions of analysis, is completed below 300 °C and occurs in two steps with the maximum weight loss at 201 °C and 294 °C (see the first derivative curve of corresponding TGA curve). When the polystyrene chains are attached to the surface of the nanoparticles via nitroxide radicals, a significant increase of thermal stability is observed (see TGA curve for Ag@PS), and the decomposition is completed above 450 °C. However, the first step of the decomposition runs at a lower temperature and maximum is located at 141 °C; this step corresponds to only 15% of weight loss. Most likely, part of the nitroxide radicals covering the nanoparticles’ surface remained not connected with polystyrene chains, and their decomposition runs at a lower temperature.

In the case of nanocomposites of Ag@PS with a mixture of PS and PVA (95%: 5%, *w*/*w*), the main decomposition step occurs above 300 °C and corresponds to 80% of total weight loss. These results confirm that the fabricated nanocomposites can be processed by thermoforming at the temperatures below 200 °C without decomposition.

The final percentage of the start mass obtained from TGA corresponds to a content of silver in the fabricated materials, and it is 62% for nitroxide-coated nanoparticles and 18% in the nanohybrids (Ag@PS) prepared from them.

As was written earlier, the developed procedure allows for obtaining the nanocomposites with different silver contents depending on the ratio Ag@PS and polymer matrix (PS/PVA). In Figure 4, the exemplified TGA curve is present for nanocomposite Ag/PS/PVA with silver content 4.6%.

Antibacterial activity of the fabricated nanocomposites was evaluated using two assays, which are commonly used for solid materials, namely the Kirby-Bauer (K-B) disc susceptibility test and the Dynamic Shake Flask (DSF) method. Because of the potential applications of the prepared materials as packaging materials, we decided to exam series of pathogenic bacteria that may be present in food and water. There are *Salmonella typhimurium*, *Escherichia coli*, *Yersinia enterocolitica*, *Campylobacter jejuni*, *Bacillus cereus*, and *Listeria monocytogenes*. Furthermore, antibacterial activity against usually antibiotic-resistant bacterial strains such as *Pseudomonas aeruginosa* and *Staphylococcus aureus* was tested.

As it turned out, all fabricated nanocomposites exhibit high antibacterial activity against all studied pathogenic strains, both Gram(−), and Gram(+).

**Figure 5:**
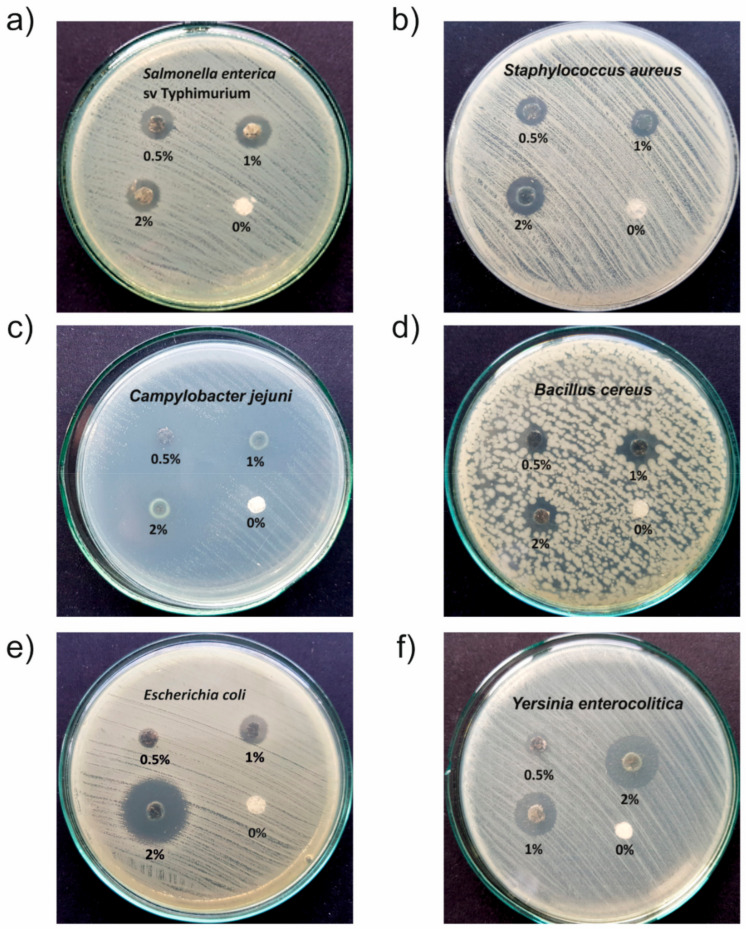
Photograph images of Kirby-Bauer susceptibility test results after 24 h of incubation at 37 °C: for Gram(-) strains (**a**,**c**,**e**) and Gram(+) strains (**b**,**d**,**f**). The sample composed of the mixture PS with PVA 95:5 (*w*/*w*) was denoted as 0% and used in all tests as a control sample.

Figure 5 shows photographic images of the results of K-B assays for selected bacterial strains. As can be seen the inhibition zones (IZs), it means the area without bacterial growth is distinctly visible for all materials, also for those with the lowest studied silver content, namely 0.5%.

The IZs determined from the performed K-B assays for all studied nanocomposites against all studied bacterial strains are presented in Table 2. As can be seen, IZs are in the range 7–34 mm, it means that the antibacterial activity of the fabricated nanocomposites is good or very good. Our results proved that the introducing of PVA as a hydrophilic component significantly increased antibacterial activity of AgNPs dispersed in a hydrophobic polymer matrix. IZ determined against *Pseudomonas aeruginosa*, and *Staphylococcus aureus* determined for the composite PS/Ag@PS without PVA is 6 mm and 4 mm at 1% of nanosilver. Whereas in the case of reported here Ag/PS/PVA, these IZs are equal to 11 mm, so are almost two times higher. The nanocomposites not containing PVA, when nanosilver concentration is below 1%, do not exhibit antibacterial activity. Thus, the introduction of a small amount of a hydrophilic polymer to the hydrophobic matrix dramatically increased the bioactivity of the composite. Most likely, it facilitates water migration inside of the polymer matrix, and this leads to the easier releasing of silver nanoparticles and silver ions.

The determined IZ values for Gram(−) bacterial strains are higher for all the studied nanocomposites in comparison with these determined for Gram(+). It can be explained by differences in the structure of their cell membranes that cause higher susceptibility of Gram(−) strains [34].

The highest susceptibility, among those studied, exhibits *Campylobacter jejuni*, the IZ value for the nanocomposite with silver content 0.5% is as high as 17 mm. It is worth mentioning that *Campylobacter* is a foodborne pathogen which is responsible for many bacterial diarrheal diseases. At the same time, antibiotic resistance of these strains is observed recently more and more frequently [35]. Thus, all the more, the fabricated nanocomposites have a strong application potential, among others, as food packaging materials.

The Dynamic Shake Flask (DSF) method was also used to evaluate the antibacterial activity of the prepared nanomaterials. This method is commonly used in the case of polymer composites and textiles. We chose three bacterial strains: *Escherichia coli* as Gram(−) representative, *Staphylococcus aureus* as Gram(+) representative, and *Yersinia enterocolitica*—a pathogen often present in food products and water causing acute infections and disease [36]. It is noteworthy that this bacterial strain can withstand low temperatures, and its growth is observed even at around 4 °C [37]. The presence of this bacteria strain may be responsible for severe problems with food storage. 

The DSF assays were performed under no-growth conditions: without nutrients, only in an aqueous solution containing phosphate buffer (0.3 M, pH = 7.3). The samples were performed in the form of pastilles made of composite (Ag/PS/PVA) with the mass 76 ± 8 mg and such content of Ag@PS to achieve 1% of silver. The buffer solution was inoculated with 10^5^ CFU ml^−1^. The pastilles were placed in the solution, and afterwards, the mixture was shaken at 200 rpm for 24 h at 37 °C. As a control sample, the pastill made of PS and PVA with a mass ratio 95:5 *w*/*w* was used. The bacterial concentration in the microbial suspension was determined by measuring optical density (O.D.) at wavelength 600 nm. The percentage survival of bacterial cells in the system was calculated according to the following equation:

(1) $$S=\frac{C_{s}}{C_{c}}\;100\%$$

where *C_s_*—surviving cell concentration in the mixture with the composite sample after an appropriate time of incubation at 37 °C, *C_c_*—surviving cell concentration in the mixture with the control sample after the same period of incubation time as *C_s_*.

The measurements of bacterial concentration were performed every hour for 6 h, and the next measurement was performed after 24 h.

**Figure 6:**
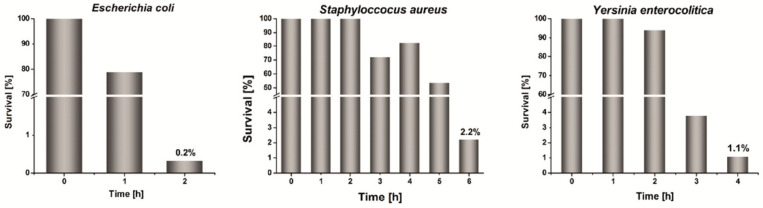
The bacterial survival under no bacteria growing conditions in DSF assay in the presence of pastilles made of Ag/PS/PVA (76 ± 8 mg) with silver content 1% and PVA content 5%.

Figure 6 displays the determined percentage survival *S*% of the studied bacterial strains vs. time of incubation in the presence of the pastilles made of Ag/PS/PVA composite containing 1% of nanosilver and 5% of PVA. In the case of all studied systems after 24 h of incubation, there were not any survived cells. In a period of only 6 h number of bacterial cells decrease rapidly under the influence of the presence of the pastilles made of the nanocomposite. The survival of *Escherichia coli* after only 2 h of incubation with Ag/PS/PVA is 0.2%. After a longer time of incubation, we did not observe any survived cells.

The survival of *Staphylococcus aureus* in the presence of the studied nanocomposite is higher and after 6 h is 2.2%, but after 24 h of incubation, we did not observe survived cells. The observed differences in susceptibility between *Escherichia coli* and *Staphylococcus aureus* are a consequence of the different structure and the composition of their cell membranes. *Staphylococcus aureus* is a representative of Gram(+) bacteria; therefore, its cell membrane is significantly thicker, and permeation through the membrane is more complicated than in the case of Gram(−) bacteria.

The survival of *Yersinia enterocolitica* is higher than *Escherichia coli* and lower than *Staphylococcus aureus*. After 4 h of incubation, in the presence of Ag/PS/PVA, the survival is 1.1%, whereas after 5 h the surviving cells were not observed.

Notably, the biocidal activity of the developed nanocomposites is significantly higher than for the analogical composites without the addition of PVA. The survival of *Staphylococcus aureus* after 24 h of incubation in the presence of pastille, made of Ag@PS and dispersed in PS with 4% silver, is almost 90% [20].

Our results revealed that the doping of polystyrene nanocomposites with a hydrophilic polymer increases their bioactivity.

The performed antibacterial assays proved that the fabricated nanocomposites could be successfully used as biocidal materials against pathogenic bacteria, both Gram(−) and Gram(+).

## 4. Conclusions

In summary, we propose a new approach for the preparation of highly bioactive and thermoprocessable nanocomposites. In the prepared materials, polystyrene-grafted silver nanoparticles (Ag@PS) play the role of bioactive fulfilment, and a mixture of polystyrene (PS) and poly(vinyl alcohol) PVA is the polymer matrix.

We also report the method allowing us to fabricate narrowly sized dispersive (5.96 ± 1.02 nm) silver nanoparticles (*N*-AgNPs) on a scale of hundreds of milligrams/several grams. Nitroxide radicals stabilize the synthesized nanoparticles, and thanks to this, they can be readily grafted with polymer chains via radical coupling during radical polymerization. Polystyrene-grafted silver nanoparticles (Ag@PS), prepared in this way, are perfectly dispersible in a mixture of PS with PVA (95:5 *w*/*w*). Such nanocomposites are thermally stable and, thus, can be thermally processed into pellets, pastilles, foils, plates, and other elements.

Kirby-Bauer (K-B) and Dynamic Shake Flask (DSF) assays showed high antibacterial activity against a series of Gram(−) and Gram(+) bacteria strains of the fabricated nanocomposites. Regarding the silver content, 2% of the biocidal activity of the nanocomposites is very high, and when the content is only 0.5%, the activity can be evaluated as being effective.

The doping of Ag/PS composites with PVA gives antibacterial activity at a low concentration of nanosilver (0.5%). Most likely, the presence of hydrophilic admixture in the nanocomposites enables water migration inside the material, which makes the releasing of silver nanoparticles and ions from the polymer matrix easier.

Due to the thermal processability and antibacterial activity at low silver content, the fabricated nanocomposites are highly promising materials for the fabrication of medical equipment such as, among others, surgical instruments, prosthesis, coatings, and dental tools.

## Figures and Tables

**Table 1:** The average molecular weights determined from SEC analysis for polystyrene that was used for the nanocomposites’ fabrication.

Sample	M_n_ [Da]	M_w_ [Da]	M_p_ [Da]	M_z_ [Da]	PDI
PSF	10,350	11,700	11,390	13,180	1.13

**Table 2:** Results of Kirby-Bauer assay for Ag/PS/PVA composites differing nanosilver content (5% *w*/*w* of PVA).

Inhibition Zones IZ [mm]			
**Gram(−)**			
	**Content of Nanosilver [% *w*/*w*]**		
	**2**	**1**	**0.5**
*Salmonella typhimurium*	12	11	11
*Escherichia coli*	22	11	7
*Yersinia enterocolitica*	14	17	8.5
*Pseudomonas aeruginosa*	13	11	7
*Campylobacter jejuni*	34	21	17
**Gram(+)**			
*Staphylococcus aureus*	12	11	10
*Bacillus cereus*	11	11	10
*Listeria monocytogenes*	10	11	10

## Supplementary Materials

The following are available online at https://www.mdpi.com/2079-4991/10/11/2245/s1, Figure S1: The photograph of the lab set employed for the *N*-AgNPs preparation taken during the synthesis. Figure S2: The elugram obtained from SEC for PSF sample. Figure S3: The molecular weights distribution in the sample PSF determined on the base of SEC analyses.
