# Supplementary material for: Silver-Polystyrene (Ag/PS) Nanocomposites Doped with Polyvinyl Alcohol (PVA)—Fabrication and Bactericidal Activity
Source: Nanomaterials (Basel). 2020 Nov 12;10(11):2245. doi: 10.3390/nano10112245 (PMC7697651; doi:10.3390/nano10112245)
Supplement: Supplementary file 1 [file nanomaterials-10-02245-s001.pdf]

nanomaterials

Supplementary Materials

Silver-Polystyrene (Ag/PS) Nanocomposites Doped With Polyvinyl Alcohol (PVA)—Fabrication and Bactericidal Activity

Anna Krzywicka and Elżbieta Megiel *

Faculty of Chemistry, University of Warsaw, Pasteura 1, 02-093 Warsaw, Poland; krzywaa95@gmail.com
*Correspondence: emegiel@chem.uw.edu.pl

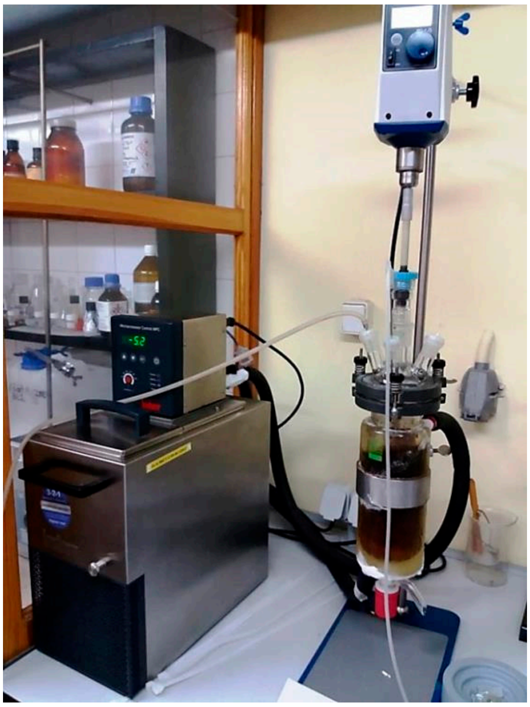

Figure S1. The photograph of the lab set employed for the N-AgNPs preparation taken during the synthesis.

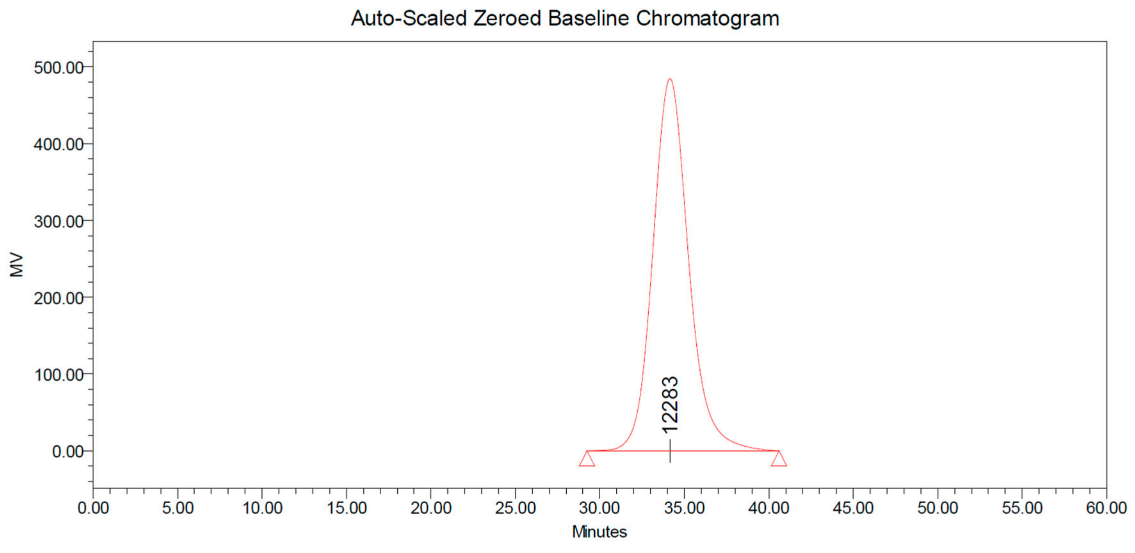

Figure S2. The elugram obtained from SEC for PSF sample.

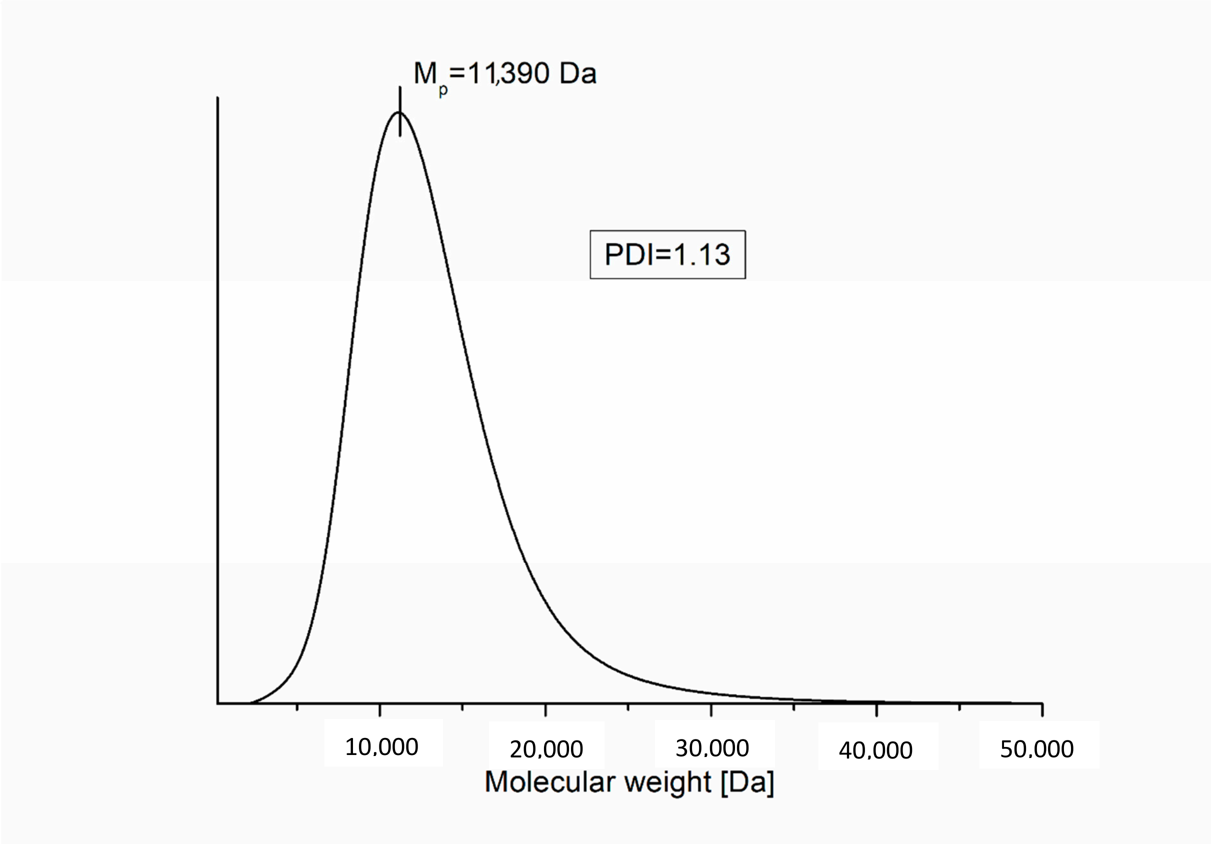

Figure S3. The molecular weights distribution in the sample PSF determined on the base of SEC analyses.
